# Helminthiasis Extraordinary

**Published:** 1897-06

**Authors:** 


### HELMINTHIASIS EXTRAORDINARY.

The following medical item appeared in *The Boston Weekly News-Letter*, January, 1717: On the Lords day Morning the sixth Currant, a strange thing fell out here, One Thomas

Smith a Sawyer about four Month ago, bought a Lusty Tall new negro, fit for his Employ, who after complain'd of something within him that made a Noise Chip, Chip, Chip; his Master sent for a Doctor, one Sebastian Henry Swetzer a German, who told him he had Worms, whereupon he gave him Physick on Wednesday; from Thursday till the Lords Day he gave him some Powders, which on the Lords Day had that effect as to cause him to vomit up a long Worm, that Measur'd a hundred and twenty eight Foot, which the negro took to be his Guts; it was almost as big as ones little Finger, its head was like a Snakes, and would receive a Mans little Finger into its Mouth, it was of a whitish Colour all full of Joynts, its tail was long and hard, and with a Microscope it seem'd to be hairy; the Negro before voiding the Worm had an extraordinary Stomach.—*Albany Medical Annals.*

